# Supplementary material for: Microsecond sub-domain motions and the folding and misfolding of the mouse prion protein
Source: eLife. 2019 Apr 26;8:e44766. doi: 10.7554/eLife.44766 (PMC6516828; doi:10.7554/eLife.44766)
Supplement: Supplementary file 1. — The parameters listed were obtained by fitting the ACFs to Equation 1 (Materials and Methods). The experiment was carried out using the W144/C199-Atto moPrP variant at pH 7, in the presence of 150 mM salt. The excitation power was measured from the counts from a calibrated photodiode placed before the main dichroic mirror. [file elife-44766-supp1.docx]

|  | **15 µW** | **30 µW** | **60 µW** |
| --- | --- | --- | --- |
| **K_1_** | 1.4 | 1.5 | 1.5 |
| **K_2_** | 1.4 | 1.4 | 1.5 |
| **K_3_** | 0.36 | 0.2 | 0.2 |
| **τ_1_ (µs)** | 1.2 | 0.8 | 0.7 |
| **τ_2_ (µs)** | 2.9 | 3.1 | 2.9 |
| **τ_3_ (µs)** | 43 | 44 | 40 |
| **τ_D_ (µs)** | 327 | 268 | 278 |
