# Supplementary material for: Microsecond sub-domain motions and the folding and misfolding of the mouse prion protein
Source: eLife. 2019 Apr 26;8:e44766. doi: 10.7554/eLife.44766 (PMC6516828; doi:10.7554/eLife.44766)
Supplement: Supplementary file 2. [file elife-44766-supp2.docx]

|  | **W144/C199-Atto moPrP** | | **W171/C225-Atto moPrP** | |
| --- | --- | --- | --- | --- |
|  | **pH 7** | **pH 7,**  **150 mM NaCl** | **pH 7** | **pH 7,**  **150 mM NaCl** |
| **K_1_** | 1 ± 0.1 | 1.5± 0.3 | 0.8± 0.3 | 0.6 ± 0.2 |
| **K_2_** | 1.1 ± 0.1 | 1.6 ± 0.3 | 0.3 ± 0.1 | 0.4 ± 0.1 |
| **K_3_** | 0.3± 0.1 | 0.3 ± 0.04 | 0.35± 0.1 | 0.3 ± 0.1 |
| **τ_1_ (µs)** | 0.5± 0.01 | 0.4 ± 0.1 | 0.4± 0.3 | 0.35± 0.1 |
| **τ_2_ (µs)** | 2.5 ± 0.2 | 2 ± 0.5 | 6 ± 4 | 2.9 ± 0.1 |
| **τ_3_ (µs)** | 50 ± 15 | 35 ± 14 | 108 ± 13 | 87 ± 10 |
| **τ_D_ (µs)** | 271 ± 16 | 278 ± 9 | 279 ± 40 | 280 ± 10 |
